# Online teaching in physiotherapy education during COVID-19 pandemic in Italy: a retrospective case-control study on students’ satisfaction and performance

**DOI:** 10.1186/s12909-021-02896-1

**Published:** 2021-08-30

**Authors:** Giacomo Rossettini, Tommaso Geri, Andrea Turolla, Antonello Viceconti, Cristina Scumà, Mattia Mirandola, Andrea Dell’Isola, Silvia Gianola, Filippo Maselli, Alvisa Palese

**Affiliations:** 1 School of Physiotherapy, University of Verona, Verona, Italy; 2 Physiotherapist, Private practitioner, Pistoia, Italy; 3 Laboratory of Rehabilitation Technologies, San Camillo IRCCS srl, Venice, Italy; 4 Department of Neuroscience, Rehabilitation, Ophthalmology, Genetics, Maternal and Child Health, University of Genoa, Campus of Savona, Savona, Italy; 5 Clinical Epidemiology Unit, Orthopedics, Department of Clinical Sciences Lund, Lund University, Entrégatan 8 Lund 22100, Lund, Sweden; 6 Department of Clinical Sciences Orthopaedics, Clinical Epidemiology Unit, Lund University, Lund, Sweden; 7 Unit of Clinical Epidemiology, IRCCS Istituto Ortopedico Galeazzi, Milan, Italy; 8 Sovrintendenza Sanitaria Regionale Puglia, Direzione Regionale Puglia INAIL, Bari, Italy; 9 Department of Medical and Biological Sciences, University of Udine, Udine, Italy

**Keywords:** Online, Distance, Digital, Education, Physiotherapy, E-learning, Student, Entry-level, COVID-19, SARS-CoV-2.

## Abstract

**Background:**

During COVID-19 pandemic, physiotherapy lecturers faced the challenge of rapidly shifting from face-to-face to online education. This retrospective case-control study aims to compare students’ satisfaction and performances shown in an online course to a control group of students who underwent the same course delivered face-to-face in the previous five years.

**Methods:**

Between March and April 2020, a class (n = 46) of entry-level physiotherapy students (University of Verona - Italy), trained by an experienced physiotherapist, had 24-hours online lessons. Students exposed to the same course in the previous five academic years (n = 112), delivered with face-to-face conventional lessons, served as a historical control. The course was organized in 3 sequential phases: (1) PowerPoint presentations were uploaded to the University online platform, (2) asynchronous video recorded lectures were provided on the same platform, and (3) between online lectures, the lecturer and students could communicate through an email chat to promote understanding, dispel any doubts and collect requests for supplementary material (e.g., scientific articles, videos, webinars, podcasts). Outcomes were: (1) satisfaction as routinely measured by University with a national instrument and populated in a database; (2) performance as measured with an oral examination.

**Results:**

We compared satisfaction with the course, expressed on a 5-point Likert scale, resulting in no differences between online and face-to-face teaching (Kruskal-Wallis ^2^ = 0.24, df = 1, *p* = 0.62). We weighted up students’ results by comparing their mean performances with the mean performances of the same course delivered face-to-face in the previous five years, founding a statistical significance in favour of online teaching (Wilcoxon rank sum test W = 1665, *p* < 0.001).

**Conclusions:**

Online teaching in entry-level Physiotherapy seems to be a feasible option to face COVID-19 pandemic, as satisfies students as well as face-to-face courses and leading to a similar performance. Entry-level Bachelors in Physiotherapy may consider moving to eLearning to facilitate access to higher education. Universities will have to train lecturers to help them develop appropriate pedagogical skills, and supply suitable support in terms of economic, organizational, and technological issues, aimed at guaranteeing a high level of education to their students.

**Trial registration:**

Retrospectively registered.

## Introduction

The ongoing Coronavirus disease 2019 (COVID-19) pandemic is still challenging educational systems worldwide. In those countries where governments decided to close educational institutions in an attempt to contain the spreading of the disease, students could not attend face-to-face activities [1]. Italy was particularly affected, with COVID-19 cases soaring already in February 2020 and lockdowns implemented as early as the 9th of March 2020 forcing all the educational institutions (from primary schools to universities) to switch to online learning [2, 3]. Within this context, the online teaching was unprecedented for different institutions, as for the entry-level Bachelor in Physiotherapy [4].

With no time for extensive training on online teaching and learning and no possibility to change the course contents, physiotherapy lecturers were faced with the challenge of effectively teaching core skills to entry-level physiotherapy students online, assuring the same competence level gained by their predecessors [5]. In the meanwhile, physiotherapy students, who were already experiencing the impact of the pandemic on their psychosocial wellbeing, had to manage the amplification of the level of negative emotions due to rapid changes in learning habits [6, 7].

Even if former systematic reviews reported that distance-online learning arouses the same satisfaction and has the same efficacy as traditional face-to-face teaching in physiotherapy [8–10], the protected experimental setting in which the included studies were conducted limits the external validity of the findings to the ongoing pandemic. A recent meta-synthesis investigated accessibility and educational methods of online education in the medical curriculum during the COVID-19 pandemic but none of the included studies investigated satisfaction and performance [11]. Thus, to the best of the authors’ knowledge, this is the first comparative study developed during COVID-19 pandemic that quantitatively evaluates students’ satisfaction and performances after attending online physiotherapy education.

Accordingly, the aims of this retrospective case-control study are: (1) to investigate students’ satisfaction and performance; and (2) to compare their degree of satisfaction and performance with those reported by students attending face-to-face courses.

## Methods

### Study design

This case-control study was developed using guidance and explanations from the Strengthening the Reporting of Observational studies in Epidemiology (STROBE) guidelines [12, 13].

 We conducted this study in compliance with the principles outlined in the Declaration of Helsinki. Written informed consent was assumed when respondents completed and submitted the survey after reading the purpose statement of the study, strategies to ensure confidentiality and privacy of the data collected. Data were fully and irreversibly anonymized by generalization of important variables [14]. Ethics approval during this pandemic was not required according to the “Ethics and data protection” regulations of the European advisory body and European Commission [14–16].

### Setting

“Advanced methodologies in musculoskeletal physiotherapy” lectures at the entry-level Bachelor in Physiotherapy have been shifted from a face-to-face to an online course in only two weeks. Before and during the pandemic, the course covers 3 main topics: clinical reasoning, analysis of pain mechanisms and evidence-based physiotherapy practice. It provides 2 ECTS (European Credit Transfer and Accumulation System), with an estimated learning workload of 50–60 h of study, of which 24 were usually fulfilled by face-to-face lectures [17].

A physiotherapist lecturer with twelve years of teaching experience in musculoskeletal physiotherapy designed and conducted the course at the University of Verona - Italy for entry-level physiotherapy students. During the weeks between the outbreak of the pandemic and the teaching of the course, the lecturer was trained by the University exclusively on the use of the online platform (how to access the system; how to record lectures; and how to upload learning materials) during a 1-hour online course. No further training on how to prepare the online teaching and how to adapt the learning content was provided.

The course was delivered online between the end of March and the end of April 2020 adopting the Panopto Secure Online Videoplatform [18]. Students’ attendance was recorded automatically by The Panopto Secure Online Videoplatform as the students accessed the lectures.

The course was organized in 3 sequential phases:

1. Power-point presentations were uploaded to the University online platform, one to briefly introduce students to the course, and the others to serve as lecture notes.
2. One week after the upload, asynchronous video recorded lectures were provided on the same platform. Each lesson lasted a maximum of 30 min [19] and included the explanation of the topic approached, a summary of the key points and a clinical case focused on the subject proposed.
3. Between online lectures, the lecturer and students could communicate through an email chat to promote understanding, dispel any doubts and collect requests for supplementary materials. Accordingly, the lecturer provided supplementary references (e.g., scientific articles, videos), also suggesting online resources (e.g., webinars and podcasts) to enhance the effectiveness of the course.

**Fig. 1:**
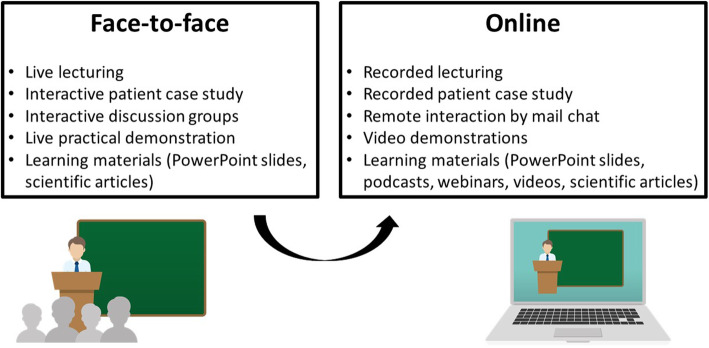
Changes in teaching strategies adopted during the transition from face-to-face to online education.

The previous five editions of the course, homogeneous in the aims and the contents, were conducted by the same lecturer. The same Syllabus developed for the face-to-face course and provided to former students was uploaded for the online edition. The admission to the oral exam was bound to 100 % attendance to the lectures both in the online and face-to-face editions. The educational strategies adopted during the transition from face-to-face to online teaching are presented in Fig. 1.

### Participants

Convenience samples for both cases and controls were considered. Students attending the course in the 2019/2020 academic year, exposed to online teaching, were considered as the online group (n = 46). Students exposed to the same course taught face-to-face in the previous five academic years (*n* = 112) were considered as a control group (face-to-face group).

### Data collection and Outcome Measures

Demographic (e.g., age and gender) and course (e.g., number of participants attending the course, number of respondents, number of passed students) characteristics were collected. The primary outcomes of interest were students’ satisfaction and performance.

The assessment of students’ satisfaction was obtained from a standardized national-established 12-item questionnaire whose compilation by students is mandatory and takes place before the final exam of each course taught in Italian universities [20]. The questionnaire covered various aspects of the course (e.g., adequacy of preliminary knowledge, balance between the study load and the number of credits assigned to this course, clarity of information on the exam structure) [20]. As a summary of students’ satisfaction, we considered the following question “*Overall, are you satisfied with the organisation and the teaching of this course*?”. Answers are allowed upon a 5-point Likert scale (“I don’t know” - value 0, “Strongly disagree” - value 1, “Somewhat disagree” - value 2, “Somewhat agree” - value 3 and “Strongly agree” - value 4) [20].

The assessment of students’ performance occurred in July of each year and was obtained through an oral exam conducted by the same teacher who delivered the lessons for both face-to-face and online courses. While the online group was assessed remotely using a real-time video-chat (Zoom), the face-to-face group conducted the exam in person at the University. The oral exam lasted a maximum of 30 min for each student and comprised open questions and a patient case study aimed at evaluating both the knowledge acquired and the ability to apply it to a clinical scenario [21]. The final grade was expressed according to the standard national metrics on a scale from 0 to 31, where 0 is the lowest value and 31 is the highest, and the minimum score to pass the course was 18/31 [22].

Satisfaction and performances shown by the online group were compared with the face-to-face groups from the previous five academic years. All data were obtained from the personal account of the lecturer, rendered available by the University of Verona (Italy) at the end of each academic year with the purpose of continuous improvement of teaching quality. Reports are divided into academic years and include anonymized students’ demographics, degree of attendance, satisfaction questionnaire responses and performances.

### Statistical methods

Descriptive statistics were used to summarize characteristics and outcomes. To report values of the dependent variables Likert scores, continuous variables were reported as medians with interquartile ranges (IQRs, 25th percentile, 75th percentile) and performances of the oral exams as mean with standard deviation (SD) or 95 %Confidence of Interval (CI). For the inferential statistics, the type of teaching (online vs. face-to-face) was considered as the independent variable. Differences in the Likert scores and the performances were explored, using the Wilcoxon rank sum test and the Kruskal Wallis test, respectively. Alpha was set at 0.05. On a preliminarily basis, given that face-to-face group included students from 2014/2015 to 2018/2019 academic years, homoscedasticity of relevant variables under study was assessed and no differences emerged (Levene’s test: Satisfaction, F_1_ = 3.60, *p* = 0.06; Performances, F_1_ = 0.41, *p* = 0.52). R software *v3.4.1* was used for statistical analysis, using ggplot2 *v3.0.0* for graphs [23, 24].

## Results

### Participants

All students of the online group (*n* = 46; 100 %) attended the course entirely. Their mean age was 24.6 (SD 2.9) years distributed as 19 females and 27 males.

**Fig. 2:**
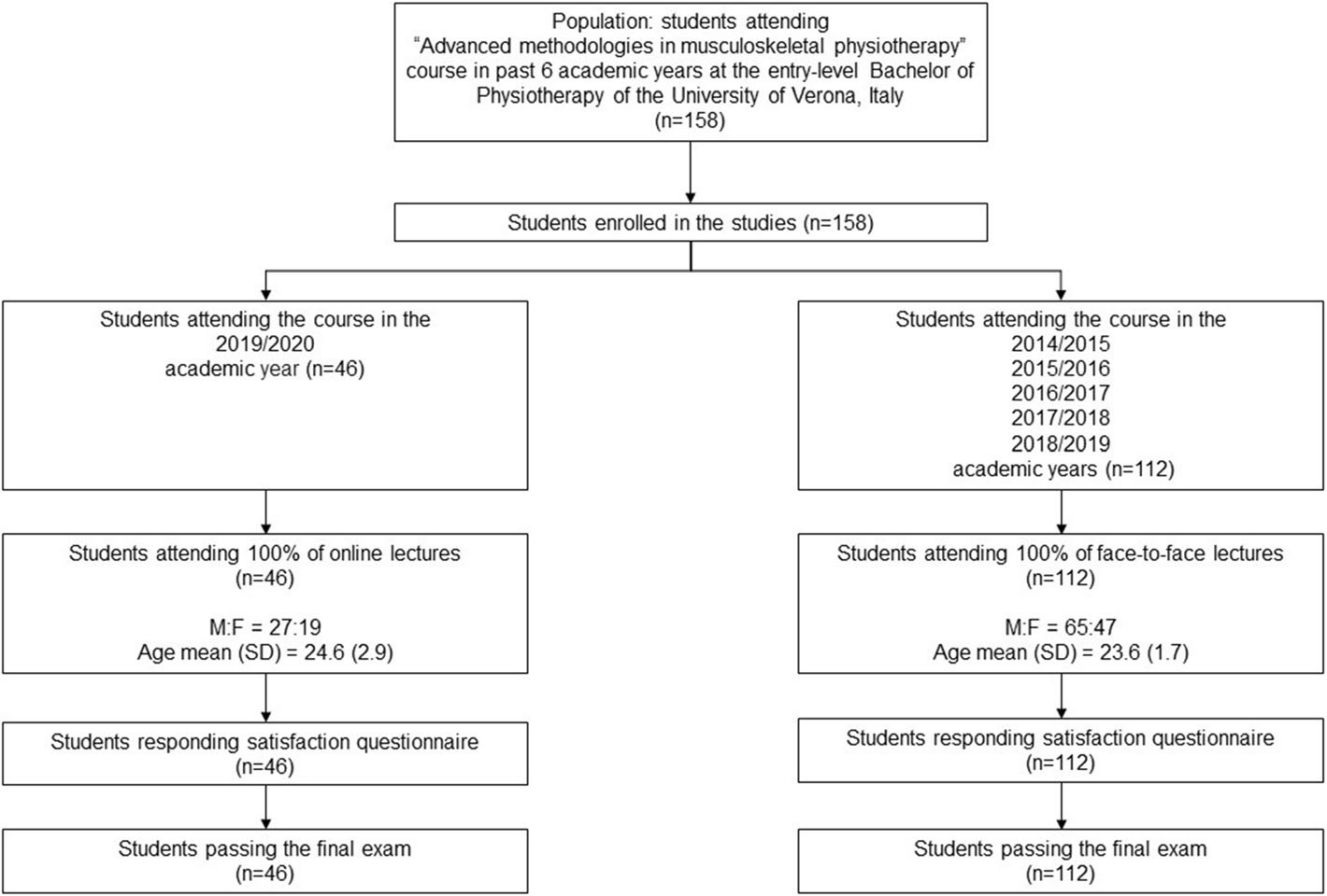
Study flowchart

Participants of the face-to-face group were 112. They all attended the course entirely (100%). Their mean age was 23.6 (SD 1.7) years, distributed as 47 females and 65 males. The graphic representation of participants is reported in the study flowchart (Fig. 2).

### Outcomes

Students attending the online course all completed the final online oral exam, with a mean performance of 29 out of 31 (95 % CI 28.2–29.7), with none failing the course. All the students responded to the University quantitative survey about satisfaction, reporting a median Likert score of 4 (Q_1_ = 3, Q_3_ = 4 [IQR = 1]).

Students attending the course face-to-face all completed the final oral exam, with a mean performance of 27.6 out of 31 (95 % CI 27.1–28.1), with none failing the course. Each of them (100 %) responded to University quantitative survey about satisfaction, reporting a median Likert score of 4 (Q_1_ = 3, Q_3_ = 4 [IQR = 1]).

There was a significant difference in the mean performances (Wilcoxon rank sum test W = 1665, p < 0.001). No difference was observed between the two groups of students in the perceived satisfaction of the course (Kruskal-Wallis Χ^2^ = 3, df = 1, *p* = 0.08) as reported in Table 1.

**Table 1:** Summary of findings

	Face-to-face group	Online group	Statistic
Satisfaction Median (IQR) 25th -75th percentile	4 (1) 3–4	4 (1) 3–4	Kruskal-Wallis *Χ*^2^ = 0.24, df = 1, *p* = 0.62
Performances Mean (SD) 95 % CI	27.6 (2.71) 27.1–28.1	29 (2.53) 28.2–29.7	Wilcoxon rank sum test W = 1665, *p* < 0.001

Abbreviations: *CI* 95 %Confidence of Interval; *IQR* interquartile range; *SD* standard deviation

## Discussion

### Key results

COVID-19 emergency pushed universities to rethink teaching methodologies, forcing teachers to learn online options to continue education and to ensure adequate educational standards [1]. To the authors’ knowledge, this is the first case-control study aimed at comparing satisfaction and performances of entry-level physiotherapy students experiencing online teaching during COVID-19 pandemic with former face-to-face students of the same Bachelor. According to the main findings, the entry-level students in Physiotherapy showed: (1) no differences in satisfaction whether they attended a face-to-face or an online course; (2) a higher performance in an online course as compared to face-to-face course.

### Interpretation

Former systematic reviews, summarizing studies performed before COVID-19 pandemic, found that levels of satisfaction and performances are similar for both distance-online and face-to-face teaching [8–10]. Our study seems to support these findings, as our online course satisfied students as the face-to-face one. These findings seem to be consistent given that the content of the course, as well as the lecturer and the type of final exam, were homogenous over the years.

The same high satisfaction was expressed by both groups suggesting that students’ needs are evolving. Higher institutions should offer flexibility in the methodologies when these are consistent with the expected learning outcomes allowing students with limited possibility to attend classes (e.g., students working) by continuing their academic career especially in countries where higher education is scarcely widespread [25]. Even if technological and set up investments for online teaching are required, studies have shown that switching to online teaching can reduce costs for students [26] which in turn can increase students numbers, especially in those countries where loans for tuitions are a major barrier to university attendance [27, 28]. However, building up a digital educational system may increase disparities towards people living in remote and rural regions, poorer social classes, and families experiencing financial difficulties due to COVID-19 induced economic crisis [26, 29, 30].

Moreover, a full eLearning Bachelor’s degree in Physiotherapy have been documented to not fulfil students’ expectations during COVID-19 [7]. First of all, online resources can act as supplementary material, but not as primary learning activities for acquiring practical skills [7]. Moreover, online-only learning has been suggested to increase distress and to hinder social interaction with peers and lecturers [7]. Even if both of these concerns can be easily related to the current uncertainty about the future [4, 6, 7], face-to-face activities have been reported more suitable to favour communication and social support also before the COVID-19 pandemic [31]. Blended teaching, combining online and face-to-face teaching, have been reported to balance benefits and drawbacks of online and face-to-face teaching [7, 8].

Regarding the higher students’ performance, our findings are in line with the growing body of evidence showing that distance-online courses can prepare students as well as face-to-face courses [8–10].

Although the difference in performance (27.6 in online vs. 29 in face-to-face group) seems to have limited practical meaning, several explanations could justify the higher performance of students in the online group. During the COVID-19 pandemic, other academic activities at universities (e.g., workshops, laboratories, clinical rotations) were suspended to ensure social distancing and physical isolation [4]. Thus, students could have spent more time studying and delving into the topics of the course. Furthermore, compared to previous years, students benefited from both different teaching strategies (e.g., PowerPoint slides, videos, podcasts, webinars) and the possibility of reviewing the recorded lecturing. This could have better matched the students’ different learning styles [32], facilitating the acquisition of knowledge for the exam. Finally, it is plausible that the evaluation of students could be less adequate, resulting in an overestimation of the performance. Indeed, an unfamiliar model of assessment (online), as well as the lack of vigilance during the exam performed at home, could lead students to academic misconduct (e.g., cheating, hint) [33]. Furthermore, the high workload to produce didactic resources, the need to perform concomitant extra academic duties (e.g., clinical service in challenging circumstances), and the difficulty to separate professional and personal activities [4, 5], could have impacted the educator and may have in turn influenced the assessment process.

### Limitations

Our study has several limitations. First, we analysed one course from a single Italian University, significantly limiting their generalizability. Moreover, the study protocol was not pre-planned but reflected circumstances imposed by the pandemic, that were worldwide unexpected [34], thus the quality of the study might have been affected. However, we decided to turn our unpreparedness into an opportunity to learn something new about didactic methodologies, and to scrutinise their effect. In this context, we could not have a synchronous control group, as Italian laws did not allow face-to-face teaching for several months [3]. In fact, one year later the declaration of the state of pandemic, all lessons are still mainly online [3]. Waiting for face-to-face teaching was not considered a feasible option, as it would have meant an unpredictable delay in students’ graduation. Thus, future studies should investigate the efficacy of online teaching using primary study design (e.g., randomized controlled trial, prospective cohort study) and including also new digital technologies (e.g., augmented and virtual reality) for educational purposes [35, 36].

Another limitation was the insufficient lecturer training in online teaching. We quickly adapted the contents of a face-to-face course to online modalities, without specific instructional design based on eLearning. If online courses in physiotherapy education will be implemented in the future, teachers will need specialised support [31]. On the other hand, teaching provided by the same experienced lecturer improved inter-groups comparability and mitigated discrepancies even if the external validity can be limited.

## Conclusions

Online teaching in entry-level Physiotherapy seems to be a feasible option to face COVID-19 pandemic, as satisfies students as well as face-to-face courses and leading to a similar performance. However, further studies should be undertaken to cumulate evidence in the field. Entry-level Bachelors in Physiotherapy may consider moving to eLearning to facilitate access to higher education. Universities will have to train lecturers to help them develop appropriate pedagogical skills, and supply suitable support in terms of economic, organizational, and technological issues, aimed at guaranteeing a high level of education to their students.

## Data Availability

The datasets used and analysed during the current study are available from the corresponding author on reasonable requests.

## Abbreviations

COVID-19: Coronavirus disease 2019
STROBE: Strengthening the Reporting of Observational studies in Epidemiology
ECTS: European Credit Transfer and Accumulation System
IQR: Interquartile range
SD: Standard deviation
M: Male
F: Female
n: Number
CI: 95 %Confidence of Interval
